# Progressive derivation of serially homologous neuroblast lineages in the gnathal CNS of *Drosophila*

**DOI:** 10.1371/journal.pone.0191453

**Published:** 2018-02-07

**Authors:** Christof Rickert, Karin Lüer, Olaf Vef, Gerhard M. Technau

**Affiliations:** Institute of Developmental Biology and Neurobiology, University of Mainz, J.-J.-Becherweg 32,Mainz, Germany; Laboratoire de Biologie du Développement de Villefranche-sur-Mer, FRANCE

## Abstract

Along the anterior-posterior axis the central nervous system is subdivided into segmental units (neuromeres) the composition of which is adapted to their region-specific functional requirements. In *Drosophila melanogaster* each neuromere is formed by a specific set of identified neural stem cells (neuroblasts, NBs). In the thoracic and anterior abdominal region of the embryonic ventral nerve cord segmental sets of NBs resemble the ground state (2^nd^ thoracic segment, which does not require input of homeotic genes), and serial (segmental) homologs generate similar types of lineages. The three gnathal head segments form a transitional zone between the brain and the ventral nerve cord. It has been shown recently that although all NBs of this zone are serial homologs of NBs in more posterior segments, they progressively differ from the ground state in anterior direction (labial > maxillary > mandibular segment) with regard to numbers and expression profiles. To study the consequences of their derived characters we traced the embryonic lineages of gnathal NBs using the Flybow and DiI-labelling techniques. For a number of clonal types serial homology is rather clearly reflected by their morphology (location and projection patterns) and cell specific markers, despite of reproducible segment-specific differences. However, many lineages, particularly in the mandibular segment, show a degree of derivation that impedes their assignment to ground state serial homologs. These findings demonstrate that differences in gene expression profiles of gnathal NBs go along with anteriorly directed progressive derivation in the composition of their lineages. Furthermore, lineage sizes decrease from labial to mandibular segments, which in concert with decreasing NB-numbers lead to reduced volumes of gnathal neuromeres, most significantly in the mandibular segment.

## Introduction

The fruit fly *Drosophila melanogaster* is an excellent model to study segmental patterning of the nervous system. Each segmental unit (neuromere) of the central nervous system (CNS) is formed by a specific set of neural stem cells (called neuroblasts, NBs), which delaminate from the embryonic neuroectoderm in a reproducible spatio-temporal pattern. Prior to delamination each NB acquires a unique identity through positional cues within the neuroectoderm (e.g. reviewed in [1]). An essential step towards understanding segmental patterning during CNS development is the identification of the individual NBs and their corresponding lineages.

The individual identity of each NB is reflected by the typical developmental time point and position of its delamination and by the combinatorial code of genes it expresses. Detailed maps have been established describing the spatio-temporal formation patterns and gene expression profiles of the NBs in the pregnathal head segments forming the brain [2–5] and of the NBs in the truncal segments forming the ventral nerve cord [6–9]. With the recently published NB maps for the gnathal head segments, which constitute a transitional zone between the brain and the truncal neuromeres of the ventral nerve cord, all neuroectodermal NBs building the CNS of the fly (except optic lobes) have been individually identified (in total 2x 567 NBs; [10]). Together, these comprehensive descriptions made it possible to systematically compare the characteristics of the NB-populations among all segments of the CNS. Embryonic neuromeres of the thoracic (T1-T3) and anterior abdominal segments (A1-A7) are formed by a rather stereotypic array of ~30 NBs per hemisegment [6,8]. While this pattern resembles the ground state (T2, which does not require input of homeotic genes [11]), NB-patterns in the terminal abdominal segments (A8-A10, [9]), the gnathal segments [10] and the brain [2–5] are derived to various degrees. This is reflected by differences in segmental NB numbers and gene expression profiles.

NBs developing from corresponding neuroectodermal `quadrants´ in different segments (exposed to similar positional cues) acquire a similar identity and are called serial homologs (e.g. reviewed in: [1,12]). The degree of similarity should be reflected by the composition of the lineages produced by serially homologous NBs. So far, comprehensive descriptions of the embryonic cell lineages only exist for NBs of the T1-A7 region. As expected, serially homologous NBs of this region give rise to similar types of embryonic lineages. Yet, several of these lineages develop segment-specific modifications affecting particular subsets of progeny cells [13–16]. As compared to thoracic/abdominal segments, NB numbers in the gnathal head segments are progressively diminished in posterior-anterior order (labial (LB) → maxillary (MX) → mandibular (MN) segment). Although all NBs formed by these segments have been shown to represent serial homologs of NBs in more posterior segments, their gene expression profiles increasingly differ from LB to MN [10].

In order to study the impact of these differences on further development we have analysed the composition of the embryonic lineages generated by NBs of the three gnathal segments using the Flybow and DiI-labelling techniques. We show that based on morphological criteria and with the help of molecular markers several of the gnathal lineages can be identified as serial homologs of lineages in more posterior segments. However, due to higher degrees of derivation of their characteristics from the ground state (T2) homologies remain obscure for most of the lineages, especially in the mandibular segment. Progressive lineage-derivation from LB to MN goes along with differences in gene expression profiles on the level of NBs. It is also reflected by a progressive reduction in lineage sizes, which contributes to decreasing sizes of neuromeres, in particular in the mandibular segment.

## Materials and methods

### Fly strains and antibodies

The following fly stocks were used: Oregon^R^ (wild type), *elav*^*C155*^-Gal4 [17], *hs-mFlp5*^*MH12*^, UAS-*FB*^*1*.*1B260b*^ [18] (both from Iris Salecker), *eve*^*RRK*^-Gal4 [19] (from Matthias Landgraf), UAS-mCD4::tdGFP, UAS-mCD8::GFP (both from Bloomington Drosophila Stock Center), *gooseberry-distal-lacZ* (from Fernando Diaz-Benjumea); *gsb-*Gal4>UAS-mCD8::GFP (from Christian Berger).

Primary antibodies used were: chicken-anti-Beta-Gal (1:1000; Abcam, #ab9361); rabbit-anti-Even skipped (1:1000) [20] (from Manfred Frasch,), mouse-anti-Even skipped (1:2; DSHB), rabbit-anti-GFP (1:250; Torrey Pines Biolabs), mouse-BP102 (1:20; DSHB), mouse-anti-Futsch (1:20; DSHB), rabbit-anti-Engrailed (1:100, Santa Cruz), mouse-anti-engrailed (1:2; DSHB), mouse-anti-ladybird (1:2, [21], from Krzysztof Jagla).

The fluorescent secondary antibodies used (all diluted 1:500, except DyLight 405: 1:250) were: anti-mouse-Alexa 647, anti-mouse-Alexa 488, anti-mouse Alexa 568, anti-rabbit -Alexa 647, ant-rabbit-Alexa 568 (all Invitrogen); anti-chicken-Cy5, anti-rabbit-Cy3, anti mouse-Cy3, anti-mouse-Cy5, anti-rabbit-FITC, anti-chicken-FITC (all Jackson Immunoresearch Laboratories), anti-rabbit-Dylight 405 (Dianova).

### Generation of Flybow clones

We combined *elav*^*C155*^-Gal4 [17]with *hs-mFlp5*^*MH12*^ [18] and crossed virgins of this stock to UAS-*FB*^*1*.*1B260b*^ [18] males.

Embryos from these crosses were collected for one hour (25°C in darkness) on an apple-juice-agar petri-dish, provided with yeast. After additional 2.5 hours at 25°C, the petri-dish was sealed with parafilm and heatshocked for 27,5 minutes in a 37°C-water bath. To stop flipase activity, the parafilm and the lid were removed and the petri-dish was set into a water-filled tray inside an incubator (14.5°C) for 38 minutes. Then the lid was closed to avoid drying of the embryos and the dish was kept for 14–15 hours in an 18°C-incubator, before it was shifted to 25°C for 2 hours to enhance fluorophore expression. Embryos were dechorionated, fixed, immunostained and flat preparations performed as previously described (e.g. [22]).

### Generation of DiI-labelled clones

DiI labelling of single neuroectodermal progenitor cells, flat preparation and photoconversion of clones were performed as previously described [13,23] in embryos homozygous for *eve*^*RRK*^-mCD8::GFP or WT. Progenitor cells were labelled at the onset of gastrulation (early stage 7), before cells of the mandibular and maxillary anlagen invaginate into the cephalic furrow. Embryos were then allowed to develop until stage 16. Flat preparations were mounted on Polylysine (undiluted) coated slides. Upon documentation of fluorescent clones, slides were turned upside down for subsequent photoconversion of the clones under a fluorescent microscope (Leica Fluovert FU).

### Documentation of embryonic lineages

Embryos carrying fluorescent DiI-clones were scanned with a Leica TCS SP2 confocal microscope using 488nm for GFP and 543nm for DiI excitation. Embryos carrying Flybow-clones were scanned with a Leica TCS SP5 using 405 or 633nm for engrailed antibody staining, 488 nm for GFP and BP102 antibody staining and 561 nm for mCherry excitation, respectively. Images were analysed using Volocity Demo 6.1.1 (Perking Elmar), and processed in Adobe Photoshop CS4, CS6 or CC2017.

Photoconverted DiI-labelled clones were viewed under a Zeiss Axioskop 2 equipped with Nomarski optic using 40x, 63x and 100x oil immersion objectives. Pictures were digitized with a Sony camera (3CCD color video camera), different focal planes were combined using Photoshop CS4, CS6 or CC2017.

### Statistical analysis of clone sizes

Although we generated 240 clones in total, in most cases the number of clones obtained for individual types of lineages was too low to show statistical significance of differences in clone sizes between segments. However, when we compared all clones from the different segments, t-Tests show that average MN clone size is different from all other segments with high significance but clone sizes between T, LB and MX are not significantly different. To increase case numbers for individual NB types we pooled MX and LB clones to compare them with the corresponding MN clones. The results of Mann-Whitney tests with these numbers are summarized in S1 Table.

## Results

### General organization of the gnathal neuromeres

**Fig 1:**
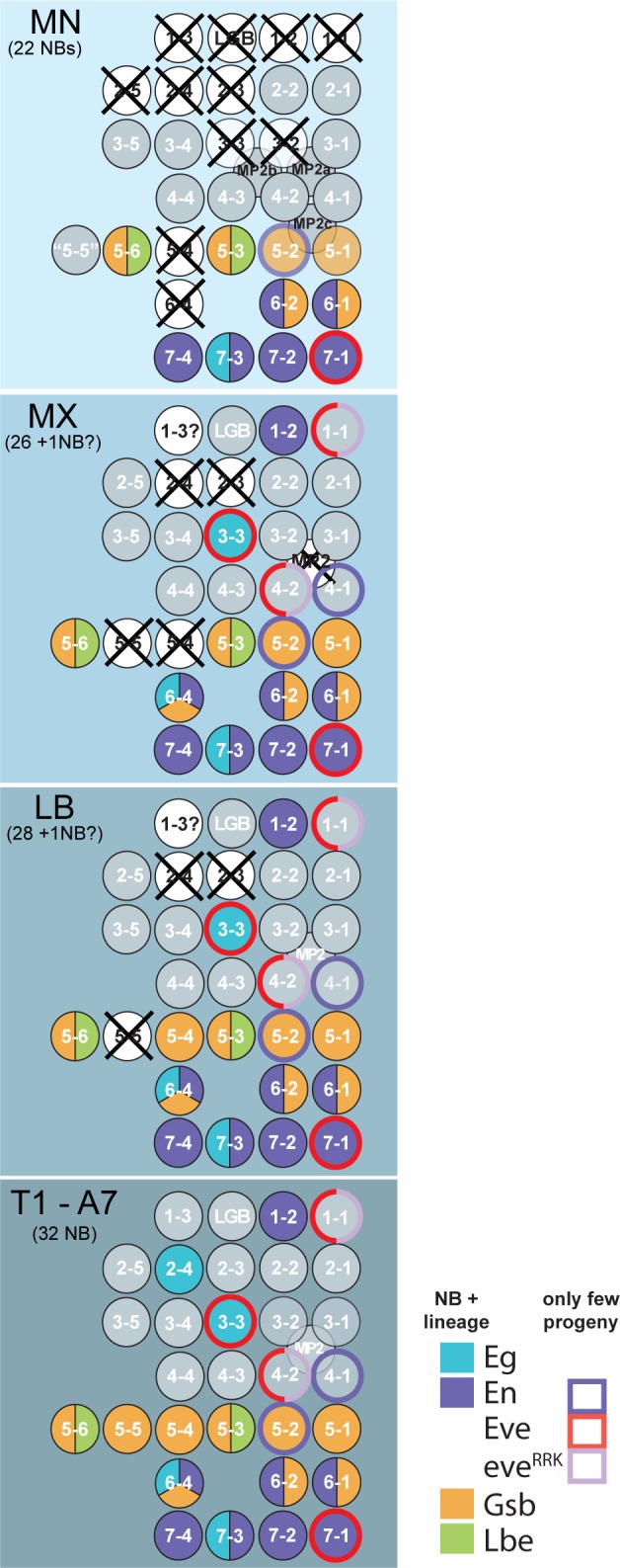
NBs present or missing in the gnathal segments and the markers used in this study. This Schematic of left hemineuromeres refers to [10] and summarizes which NBs are formed and which are missing (crossed out) in the different gnathal segments. NB-populations in T1-A7 correspond to the ground state pattern. In addition, NBs are colour coded to show the markers that were used in this study; these markers either stain the entire lineage or only a specific set of progeny cells (indicated by outer circles). MN: mandibular segment; MX: maxillary segment; LB: labial segment; T: thoracic segments; A: abdominal segments. LGB: longitudinal gliaoblast; Eg: *eagle;* En: *engrailed;* Eve: *even-skipped;* eve^RRK^: *eve*^*RRK*^-Gal4; Gsb: *gooseberry*; Lbe: *ladybird early*.

**Fig 2:**
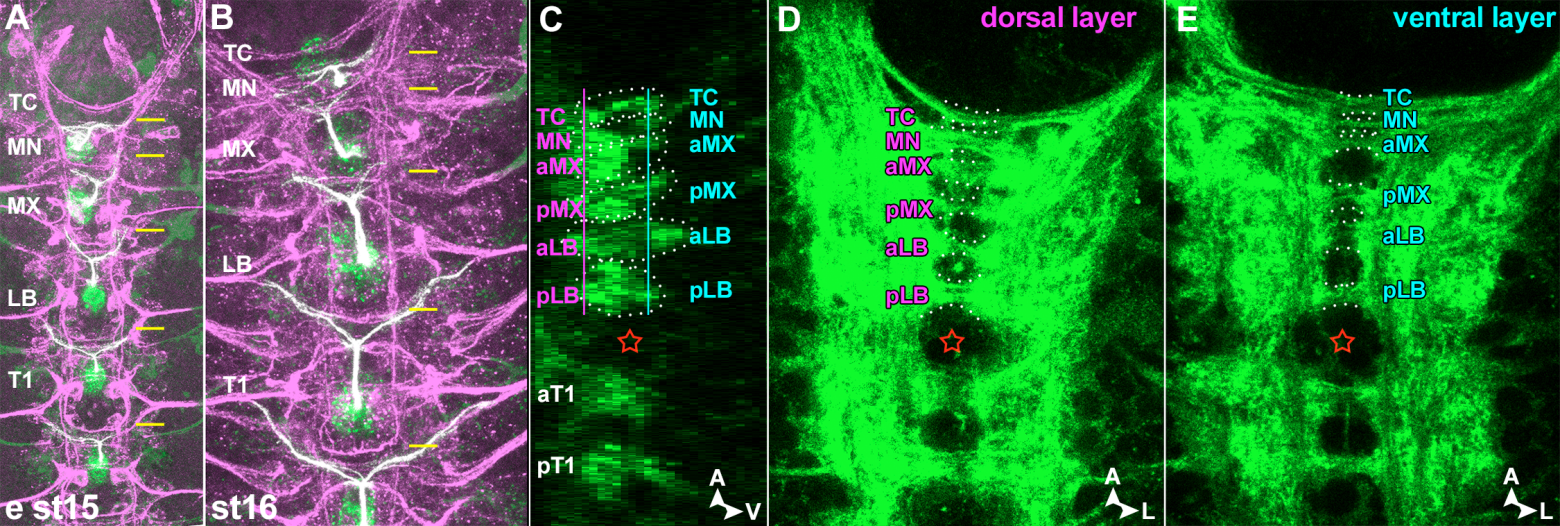
Sizes and neuropil structure of gnathal neuromeres. **(A, B)** Horizontal views on early stage 15 (**A**) and stage 16 (**B**) ventral nerve cords stained with 22C10 (antiFutsch; magenta) and manually pseudocoloured to emphasize VUM neuron position. VUM cell bodies (green) lie mid-ventrally in the posterior half of each segment and send axons (green and magenta = white) anteriorly into the anterior commissure where they bifurcate and turn laterally. Yellow lines mark the anterior margin of the anterior commissures. Thoracic (T1) and labial (LB) segments share similar sizes while the maxillar segment (MX) is clearly smaller, and the mandibular segment (MN) is only about half the size of MX. **(C–E)** Mid-sagittal (**C**) and horizontal (**D, E**) views on a stage 16 ventral nerve cords stained with BP102. The focal planes of **D** and **E** are indicated by the magenta and cyan lines in **C.** Anterior to the larger gap between thoracic and gnathal commissures (red star) BP102 shows six commissures: two in the LB, two in the MX (anterior (a) and posterior(p), respectively), one in the MN and one belonging to the tritocerebrum (TC). All commissures show more dorsal (magenta layer) and more ventral bundles (cyan layer).

Organization of the three gnathal segments (labial (LB), maxillary (MX) and mandibular (MN) segment) gradually differs from the ground state in anterior direction. The segmental populations of neuroblasts (NBs) are progressively diminished, i.e. from LB (28 NBs per hemisegment, hs), MX (26 NB / hs) to MN (22 NBs / hs) [10] (see also Fig 1). Compared to the thoracic ground state (32 NBs / hs; [8]) NBs are lacking preferentially in the anterior compartment of each segment. In the MN, which forms the smallest neuromere (Fig 2A and 2B), this has an impact on commissure formation. While in the LB and MX, similar to thoracic/abdominal neuromeres, an anterior and a posterior commissure are formed in each segment, the MN develops only one commissure (Fig 2). It´s composition may largely correspond to the posterior commissure, considering the reduction of the anterior MN neuromere compartment. However, it likely also carries fibres from the residual anterior compartment. In addition the mandibular commissure initially forms a fascicle with the tritocerebral brain commissure on the ventral side of the foregut, which then separate during embryonic stages 13 to 15 into two distinct commissures ([24–27]; Fig 2).

### Tracing the lineages of gnathal neuroblasts

**Fig 3:**
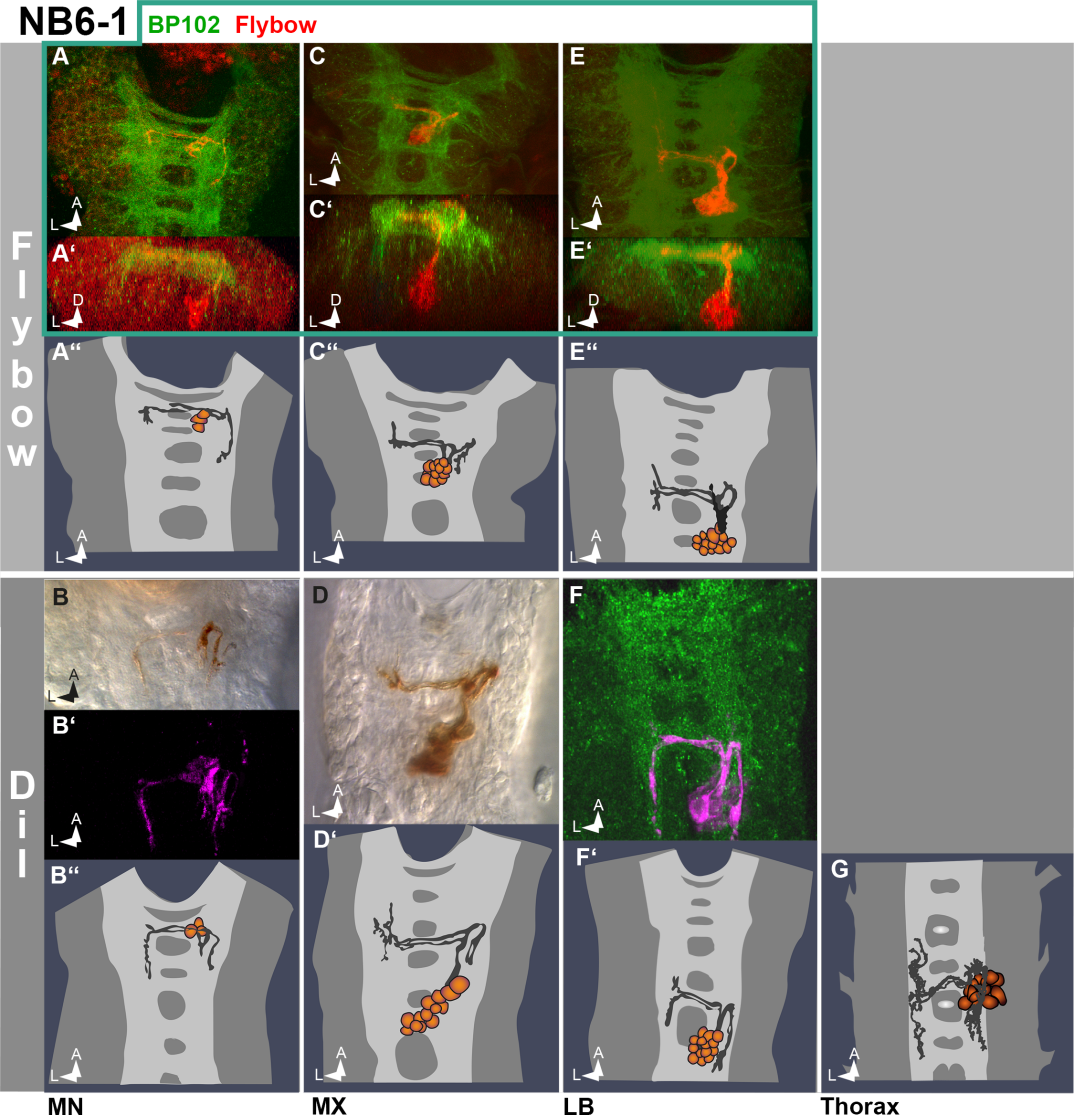
NB6-1 lineage in gnathal segments. Frontal (in **A´**,**C´** and **E´**) and horizontal (all other) views on late embryonic ventral nerve cords (late stage 15 to late stage 16) showing Flybow clones (**A**, **C** and **E**) and DiI clones (**B**, **D**, **F** and **G**). Semischematic drawings are shown in **A´´, B´´, C´´, D´, E´´, F´** and **G**). Serially homologous NB6-1 lineages are clearly identifiable based on their characteristic projection pattern and cell body positions: ventral and medial cells send a prominent contralateral fascicle dorsally through the posterior commissure and an ipsilateral bundle posteriorly. Yet, the number of cells is significantly reduced in MN (see S1 Table). Note comparable clone sizes between corresponding Flybow- and DiI-labelled clones (A´´vs. B´´, C´´vs. D´, E´´vs. F´), demonstrating that Flybow labels the entire lineage of this rather late delaminating NB.

**Fig 4:**
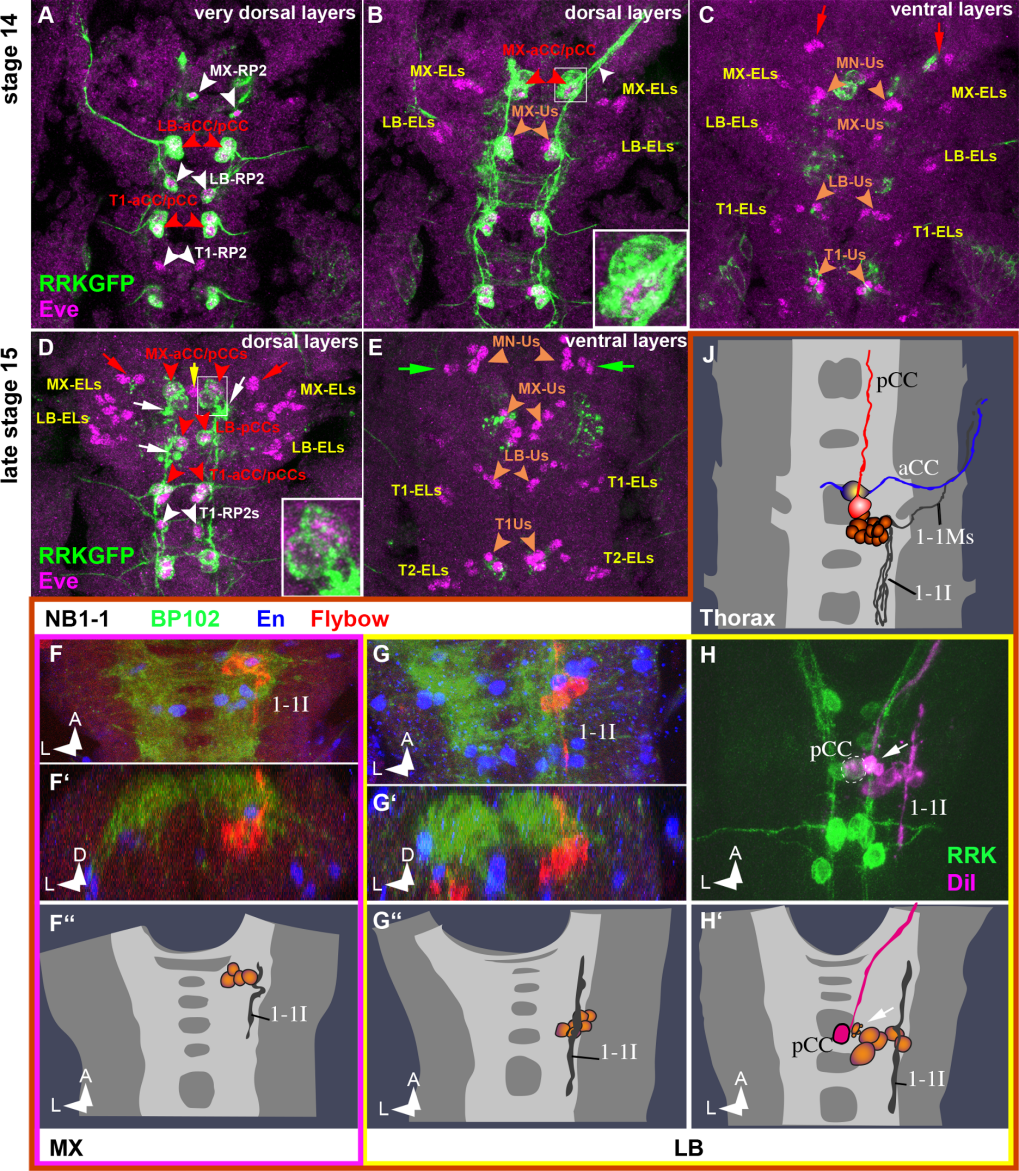
Eve/EveRRK expression and the NB1-1 lineage in gnathal segments. (**A–E**) **Eve/EveRRK expression.** Horizontal views of different focal planes of anti Eve (nuclear, magenta) and RRK-GFP (cell membranes, green) double stainings. (**A**, **B**) Until stage 14 the MX and LB segments show the full set of three cells per hemineuromer known to express the RRK-driver: aCC and pCC (stemming from NB1-1, red labels and arrowheads) and RP2 (stemming from NB4-2, white labels and arrowheads), typically located in dorsal positions. (**B**, **C**) The more ventral Eve-expressing cells comprise the Eve-lateral cells (ELs, stemming from NB3-3, yellow labels) and the 3 to 5 U-neurons per hemineuromer (Us, stemming from NB7-1, orange labels and arrowheads). Note that the U-neurons are the only identifiable Eve-expressing cells in MN. However, there is a pair of cells of unknown origin located more anterior on either side (red arrows in **C** and **D**). (**D**, **E**) At later stages all gnathal RP2s and the labial aCC undergo apoptosis, indicated by cell debris in dorsal positions (white arrows in **D)** resulting in a total of only 6 RRK-positive cells in the gnathal segments: the two pCCs in LB and two pairs of aCC/pCC in MX (see the four most anterior red arrowheads in **D**). The number of non-identified Eve cells increases: one unpaired mid-dorsal cell (yellow arrow in **D**) and one ventral cluster per side at the border between MN and MX (green arrows in **E**). Interestingly, in MX the more dorsal cell of the aCC/pCC pairs (in all other segments this is aCC) is RRK- but not Eve-positive (insets in **B** and **D;** relative a/p position appears shifted due to morphogenetic movements between stages). In addition, the aCC motorprojection does not leave the MX neuromere but projects alongside pCC anteriorly within the connective (white arrowhead in **B**). (**F–J) NB1-1 lineage**. Frontal (**F´, G´**) and horizontal (all other) views of NB1-1 clones (semischematic drawings in **F´´, G´´, H´** and **J** and composed confocal stacks in all other images), labelled with Flybow (**F** and **G**) or DiI (**H** and **J**). In thoracic segments (**J**) the NB1-1 lineage shows the characteristic dorsal pair of identified first born cells: the ipsilaterally projecting motorneuron aCC (blue projection) and the anteriorly projecting interneuron pCC (red projection). In addition, there is a cluster of cells directly ventral to the neuropil that send projections posteriorly in a lateral and dorsal position within the connective (interneurons 1-1I) and also include an ipsilateral motorprojection (1-1Ms). In the labial segment both motorneurons (i.e. aCC and 1-1Ms) are not present (**G** to **H´**). While it is obvious that aCC dies (see cell debris at white arrows in **H, H´** and **D**) the second motorneuron does not appear to be formed. The lateral interneuronal projections (1-1I) are present and extend posteriorly and anteriorly. Note that, although pCC is clearly present in that segment (“pCC” in H and H´ and “LB-pCC” in D) the labial Flybow clone lacks pCC (no medial cell with long anterior projection is present in G to G´´) because the firstborn cells remain unlabeled. Accordingly, also maxillar NB1-1 Flybow clones do not show aCC and pCC (no medial cells with long anterior projection are present in **F** to **F´´**), although both are formed (see “MX-aCC/pCC” and inserts in **B** and **D).** Unfortunately we did not generate NB1-1 DiI clones in MX.

We adopted the Flybow system to efficiently and arbitrarily mark embryonic clones with a fluorescent tracer (mCherry) [18,28]. As a driver line we used *elav*-GAL4 [17], which is constantly expressed in all neurons of the CNS [29,30] and transiently expressed in embryonic glial cells and NBs [31]. Flipase activity was induced during stages 6 to 8, i.e. before delamination of the first NBs. However, in Flybow clones recombination may take place after first divisions of NBs rendering their first progeny cells unlabelled. This is mainly the case for NBs delaminating early, whereas most lineages deriving from later born NBs appeared to be entirely labelled (see also [32]). We confirmed this for a number of clones by applying the DiI-labelling technique, which allows complete staining of embryonic lineages [23]. For example, while Flybow labelled the entire lineage of the late delaminating NB6-1 (compare the similar clone sizes of DiI- and Flybow-labelled clones in Fig 3), the early delaminating NB1-1 is lacking the marker in its first progeny cells (see Fig 4F–4H´). Yet, the DiI-technique is challenging and time-consuming, and those regions of the gnathal neuroectoderm (part of mandibular and maxillary segment), which at the onset of gastrulation invaginate into the cephalic furrow, are hardly accessible for the dye filled capillary. Therefore, our analysis of gnathal NB-lineages is mainly based on the evaluation of Flybow-labelled clones.

Labelled NB-lineages were analysed at late stages (early stage 15 to late 16) when all embryonic progeny cells were generated and have reached an advanced stage of differentiation. The neuropil scaffold (commissures, connectives and nerve routes labelled by BP 102) and engrailed-staining served as landmarks to which the individual lineages were related. For each clone 1) the segmental affiliation, 2) the number and 3) the types of progeny cells, 4) the 3D-position of their cell bodies in the cortex, 5) the neuronal fibre projections in the neuropil and in some cases 6) the expression of specific molecular markers was established. Based on these criteria individual types of gnathal NB-lineages and by comparison with the known lineages in the thoracic/abdominal segments [13–15] putative serial homologs were identified.

### The composition of gnathal neuroblast lineages

In total, we labelled 240 clones in the gnathal neuromeres. 114 of these were located in LB, 90 in MX, and 36 clones in MN (Table 1). Lineages of the CNS midline precursors were not included in this study. While in LB 91% of clones were identifiable (based on the criteria mentioned above), their proportion was 82% in MX and only 50% in MN (see Table 1 for details).

**Table 1:** Types and sizes of the labelled clones.

	MN			MX			LB		Thorax
clone type	times labelled	clone size		times labelled	clone size		times labelled	clone size	clone size
NB1-1	X			4	5–8		14	6–8	10–16
NB1-2	X			0			7	10–14	16–24
NB2-1	0			2	3–8		4	3–5	5–10
NB2-2	2	6–7		2	11–14		4	4–12	12–14
NB2-5	X			1	10		3	8–9	15–20
NB3-1	0			4	4–10		3	11–13	12–15
NB3-2	X			0			2	9–12	10–18
NB3-3	X			3	8–11		2	8	10–13
NB3-3 or 4–4				5	6–11				
NB3-5	0			5	10–20		7	15–25	19–24
NB4-1	0			6	6–16		8	10–20	12–18
NB4-1 or 5–2				2	8–15		1	20	
NB4-2	0			2	10–15		4	8–14	10–16
NB4-4	0			3	7–10		0		8–11
NB5-2	2	8–9		6	10–20		6	14–20	17–26
NB5-3	1	8		2	11–12		3	6–12	9–15
NB5-6	0			8	4–15		3	12–20	15–19
NB6-1	2	3		3	11–19		5	10–16	10–16
NB6-2	1	5		2	15–20		5	16–20	8–16
NB6-4	X			1	4		0		7–9
NB7-1	0			7	6–20		7	10–20	16–22
NB7-2	6	5–9		3	9–17		4	8–14	8–14
NB7-3	3	2		3	2–3		9	3–5	3–5
NB7-4	1	3		0			1	10	13–18
MP2	0			0			2	2	2
NN	12	3–12		11	5–18		10	5–15	
clone type A				5	6–12				
clone type B	3	9–10							
clone type C	3	6–11							
**percentage of non-identified clones**	**50**	** **	** **	**17.8**	** **	** **	**8.8**	** **	
									
**total number**	**36**	** **	** **	**90**	** **	** **	**114**	** **	
**median clone size**	** **	**6.4**	** **	** **	**10.2**	** **	** **	**11.3**	**13.2**

**Table 1** lists all labelled clones in the different gnathal neuromeres (columns MN, MX and LB) and as a reference the thoracic clone sizes taken from [13,15](column: Thorax). The median clone size drops from 13.2 in the thorax to 11.3 cells in LB, 10.2 cells in MX and 6.4 cells in MN. T-Tests show that average MN clone size is different from the other segments with high significance (p < 0,0001 for both), but clone sizes between T, LB and MX are not significantly different. The degree of clonal derivation is also reflected by the percentage of non-identified clones rising from 8.8% in LB to 17.8% in MX and 50% in MN. Red fields with X mark lineages, that do not exist in MN.

NN: non identifiable clones; clone types A-C correspond to clone morphologies we found more than once but were not able to identify (see S1 Fig).

In the following we describe gnathal NB-clones showing characteristics that allowed to identify them as serial homologs of lineages in more posterior segments and, thus, to assign them to specific NBs.

The clones shown in Fig 3 resemble the NB6-1 lineage in thoracic/anterior abdominal segments [13]. They consist of a cluster of interneurons located in the ventral cortex close to the midline, which develop similar ipsi- and contralateral fibre projections. However, the number of cells is significantly reduced in MN (in both Flybow- and DiI-labelled clones), compared to their more posterior homologs.

**Fig 5:**
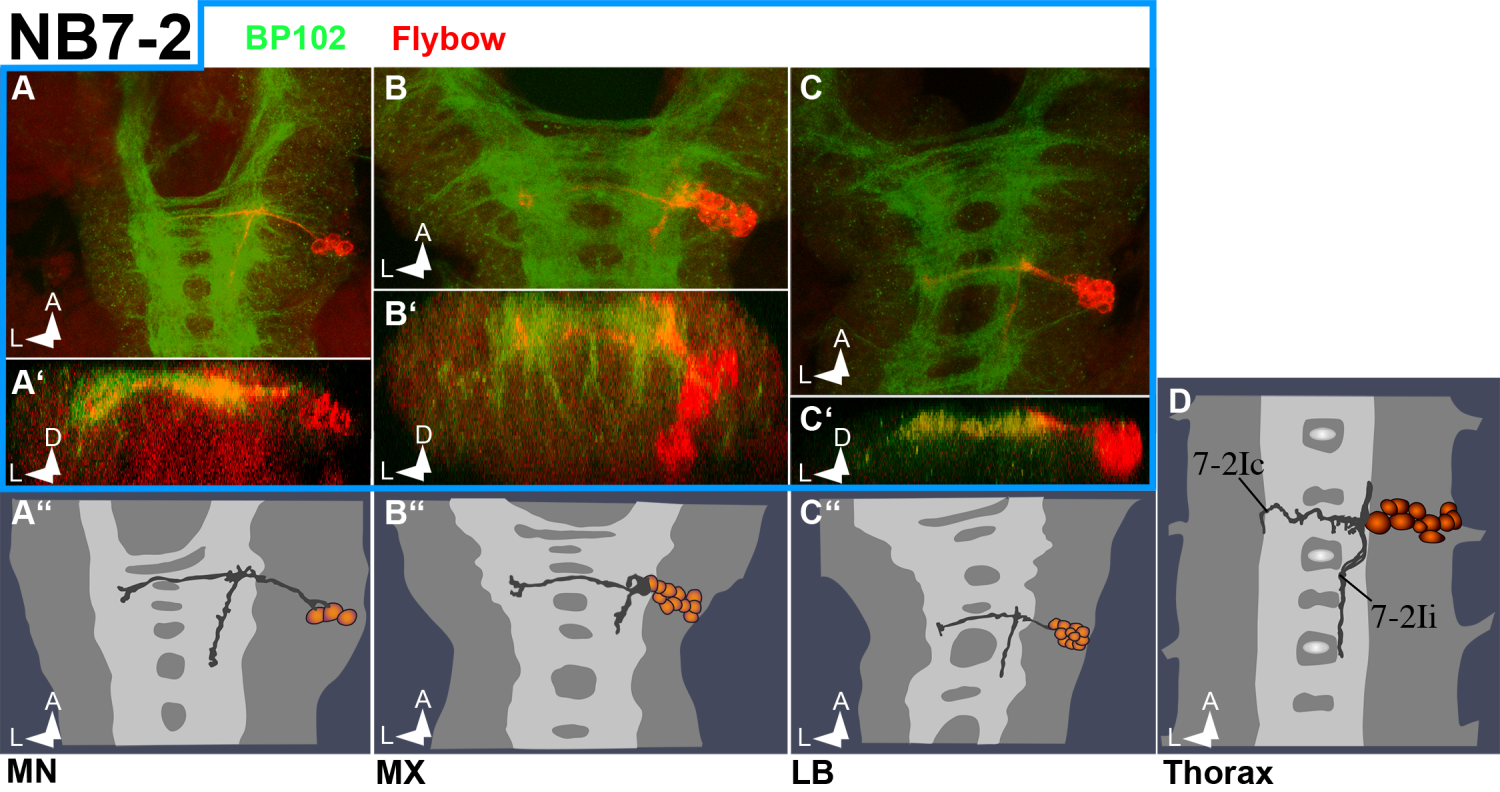
NB7-2 lineage in gnathal segments. Frontal (**A´, B´** and **C´**) and horizontal (all other) views of Flybow clones (semischematic drawings in **A´´, B´´, C´´** and **D,** and composite confocal images in all other) resembling NB7-2 lineage. Fibre projections show very little modulation across the segments: an ipsilateral descending interneuronal fascicle in a dorsal position within the connective and a contralateral bundle through the posterior commissure. Cell numbers are significantly reduced in MN (see S1 Table).

Fig 5 shows the gnathal homologs of the NB7-2 lineage as revealed by the position of their cell bodies and projection pattern. Again, the number of these interneurons is clearly reduced in MN.

**Fig 6:**
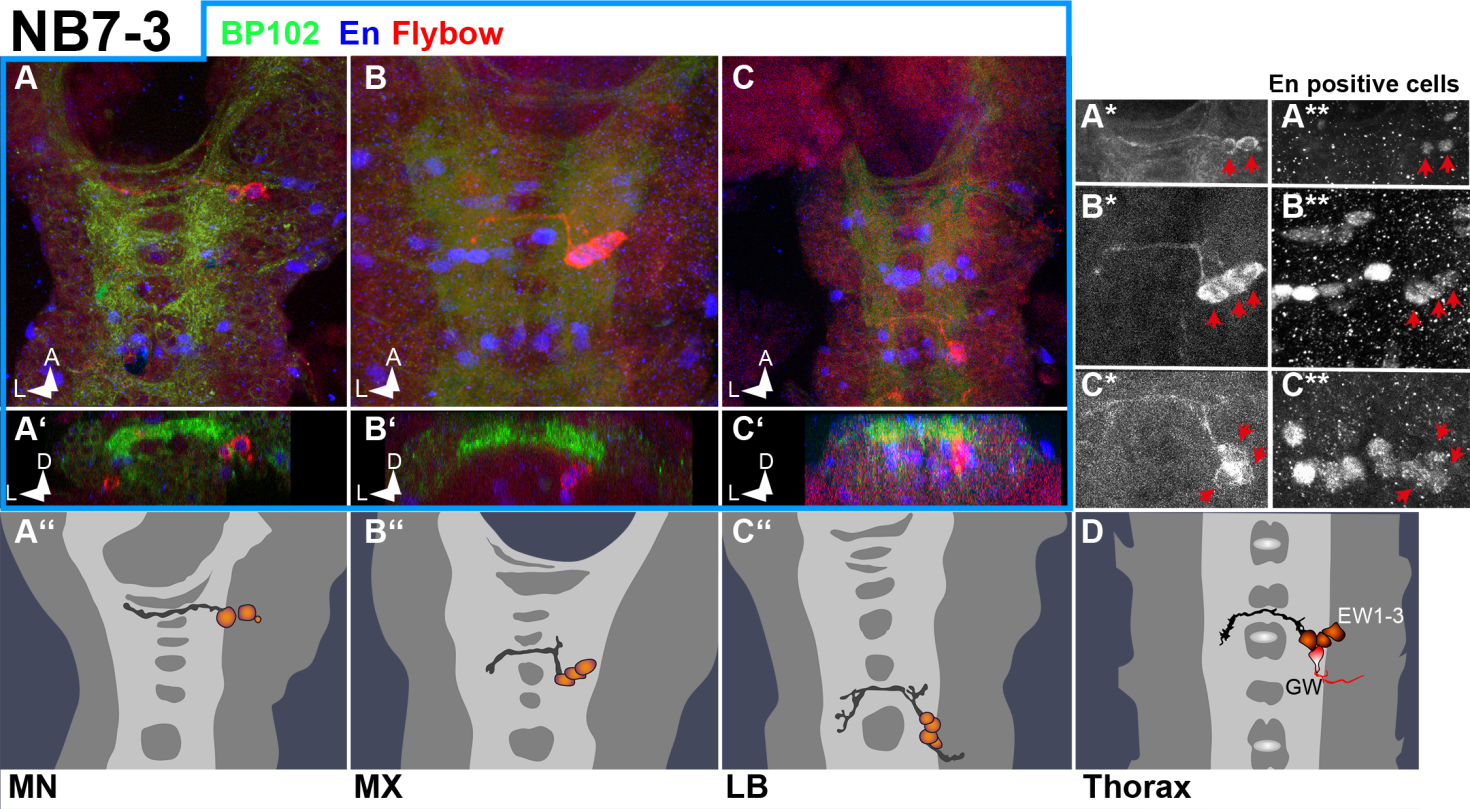
NB7-3 lineage in gnathal segments. Frontal (**A´, B´ and C´**) and horizontal views (all other) of Flybow clones (semischematic drawings in **A´´, B´´ C´´** and **D**, and composite confocal images in all other) resembling NB7-3 lineage. In labial segments (**C–C´´**) NB7-3 lineage is identical to thoracic segments (**D**), showing the contralaterally projecting EW 1–3 interneurons and the ipsilaterally projecting efferent GW neuron located directly ventral to the neuropil. In MX (**B–B´´**) the GW, and in MN (**A–A´´**) the GW and one of the EW neurons are missing. Note that the EW neurons express Engrailed in all segments (red arrows in A* and A** (MN), B* and B** (MX) and C* and C** (LB).

Clones in Fig 6 appear to be serially homologous to the NB7-3 lineage. In thoracic and anterior abdominal segments this lineage consists of three interneurons with contralateral projections and one neuron (GW neuron) with an efferent ipsilateral projection ([13,33]; Fig 6D). The same composition is seen in LB, whereas the lineages in MX and MN are lacking the GW neuron and, in addition, in MN one of the interneurons is missing. This is in agreement with the expression-pattern of the molecular markers Engrailed (labelling the GWs; Fig 6A*–6C**) and Eagle (labelling the entire NB7-3 lineage; [33–35] in gnathal segments. In segments T3-A8, the GW neuron undergoes cell death in the late embryo, but survives in T1,T2 [36]. In the gnathal lineages GW survives in LB, but undergoes cell death or is not formed in MX and MN.

**Fig 7:**
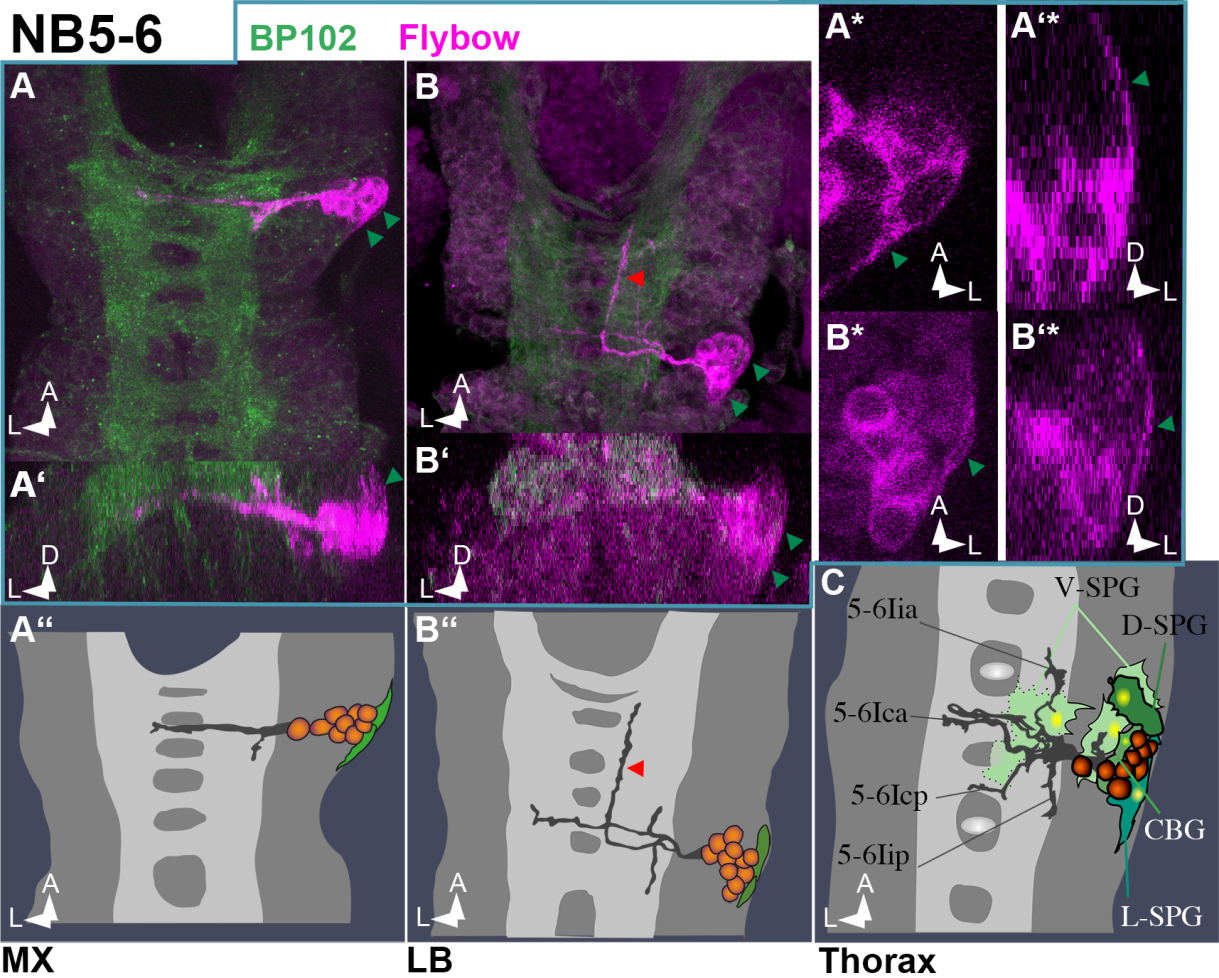
NB5-6 lineage in gnathal segments. Frontal (A´, B´, A´* and B´*) and horizontal (all other) views of Flybow clones (semischematic drawings in **A´´, B´´ and C** and composite confocal stacks in all other images) resembling NB5-6 lineage. In thoracic segments (**C**) NB5-6 is a neuroglioblast that gives rise to about 5 glial cells and several interneurons that project through both commissures and form short axons along the ipsilateral connective in both directions [14]. Although Flybow clones generated with an Elav driver are expected to show only low hue-expression in glial cells, the clones reveal a couple of cells at the cortex margin with glial-like shape (green arrowheads in the confocal images and green cells in **A´´ and B´´**); A* to B´* zoom into single layers of the image stacks in **A** and **B** to show the typical morphology of subperineural glial cells: in a location directly at the margin of the nerve chord with long and thin protrusions that cover the cortex. The projection pattern of the LB lineage largely resembles the thoracic one, but shows an additional long ipsilateral projection in anterior direction (red arrowhead in **B** and **B´´)**. In MX the lineage shows reduced projections.

Serial homologs of neuroblast NB5-6 have been uncovered in all three gnathal segments [10]. We identified a NB5-6 lineage in MX and LB (Fig 7A and 7B´´), which, like it´s thoracic/abdominal homolog [14,37] Fig 7C), consists of interneurons and glial cells. In LB the neuronal projection pattern is similar to thoracic clones, but shows an additional ascending ipsilateral projection (red arrowhead in Fig 7B and 7B´´). Interestingly, this unique projection has been described for labial NB5-6 clones in 3^rd^ instar larvae (see Fig 8 in [38]). The number of fibre bundles is considerably reduced in MX. We were not able to identify a corresponding clone in MN.

**Fig 8:**
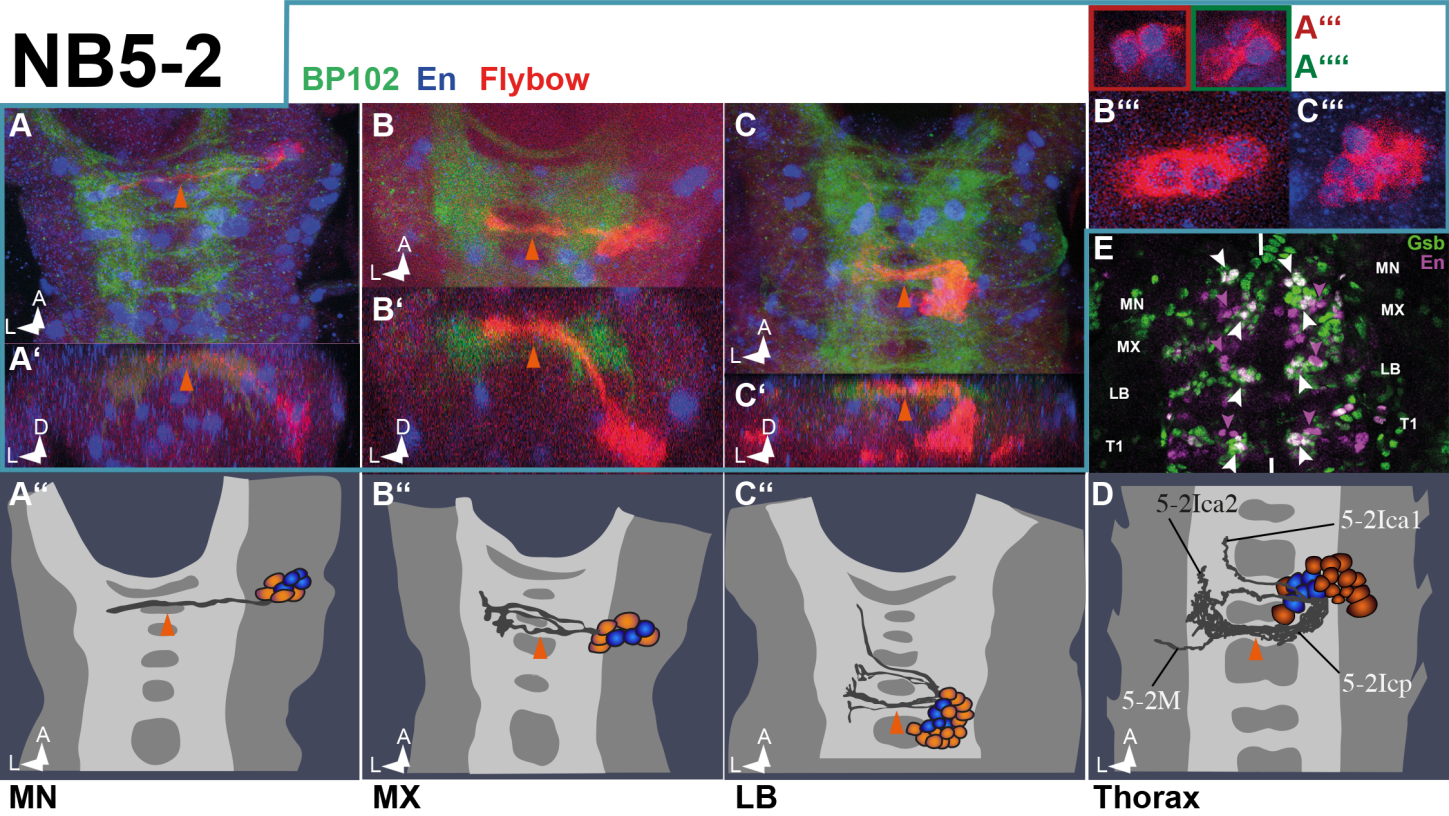
NB5-2 lineage in gnathal segments. (**A–D)** Frontal (**A´, B´, C´, A´´´´**) and horizontal (all other) views of NB5-2 clones (semischematic drawings in **A´´, B´´, C´´** and **D** and composed confocal stacks in all other images). In abdominal and thoracic segments (**D**) NB5-2 generates a large clone with more than 20 cells that lie in a ventral and medial position. It is one of the few clones that send projections via both segmental commissures (see also MX and LB). In MN the cells of the NB5-2 lineage lie ventrally at the anterior border of the VNC sending a single projection through the commissure dorsally (**A** and **A´**) similar to 5-2Icp (red arrowheads in A–D). As we used Engrailed (En) as an obligatory landmark staining we noticed that 3 to 5 NB5-2 progeny cells express En at late stages (blue cells in **A´´-C´´, A´´´-C´´´** and **D**). (**E)** The En-positive NB5-2 cells are the only ventral cells that strongly co-express Gooseberry (Gsb) (white arrowheads). Note the pair of NB4-1 En-positive cells (magenta arrowheads) that lie antero-laterally to the NB5-2 cells in MX, LB and T1 (NB4-1 not present in MN).

Further clones were identified with the help of molecular markers. We noticed that in the NB5-2 lineage (Fig 8) 3 to 5 progeny cells start to express Engrailed (En) at later stages (the NB itself being En-negative; Fig 8A´´´–8C´´´). This is similar to En- negative NB4-1, which gives rise to two En-positive cells [39]. Since row 5 NBs also express Goosberry (Gsb) [8,40] NB5-2 progeny cells are the only ventral cells that express both markers (Fig 8E, white arrowheads). While serial homology of NB5-2 clones is quite obvious from morphological criteria in MX and LB (Fig 8B and 8C´´), the En/Gsb co-expressing cells also allowed identification of the NB5-2 lineage in MN, despite of its rudimentary composition and surprisingly anterior position of the cell bodies. Corresponding Flybow clones show only a single fibre bundle projecting dorsally through the commissure (Fig 8A), which most likely represents the 5-2Icp fascicle (Fig 8D) of its posterior homologs. NB5-2 is one of the cases where the noticeable reduction of clone size in MN also can be shown to be significant (see S1 Table).

**Fig 9:**
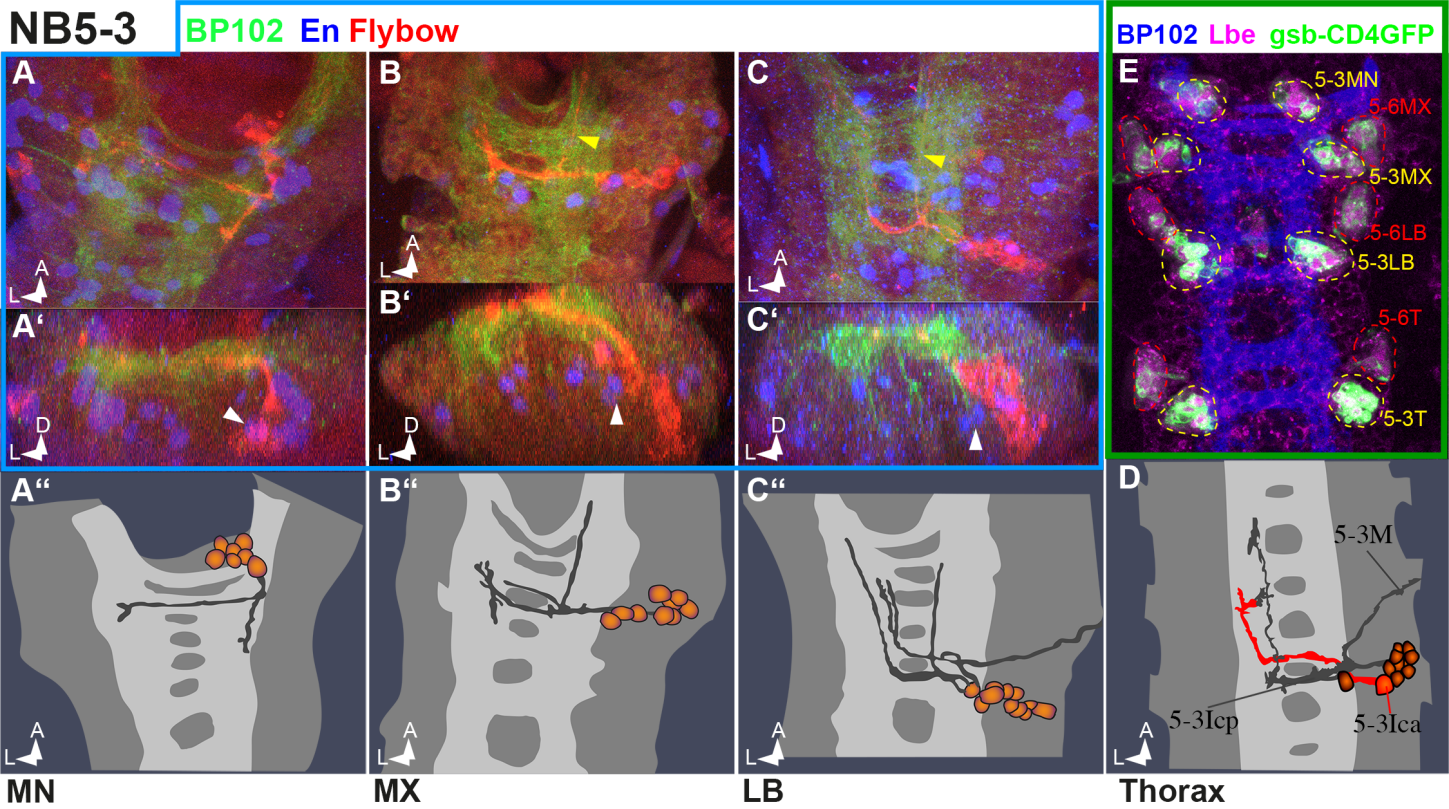
NB5-3 lineage in gnathal segments. (**A–D)** Frontal (**A´, B´, C´**) and horizontal (all other) views of NB5-3 clones (semischematic drawings in **A´´, B´´, C´´** and **D** and confocal compositions in all other images). In thoracic and abdominal segments (**D**) the NB5-3 cell cluster lies in a ventrolateral position. It contains an ipsilateral motoneuron and interneurons projecting contralaterally through both commissures (according to [13,14]). In LB the lineage looks very similar but has an additional ipsilateral interneuronal fascicle turning anterior (yellow arrowhead in **C)**. In MX the motoneuron is missing but projections through both commissures and the additional ipsilateral ascending projection (yellow arrowhead in **B**) are present. In MN the lineage generates one contralateral and an ipsilateral descending projection. Note the presence of NB5-2 derived EN-positive cells located medially to the NB5-3 cell cluster in all segments (white arrowheads in **A´ –C´**). **(E)** Late stage 15 VNC stained against Ladybird (Lbe, magenta), BP102 (blue) and *gooseberry*-GAL4>UAS-CD4::tdGFP (gsb-CD4GFP, green). The image is a composite of selected ventral Gsb and Lbe areas and a single more dorsal BP102 layer to allow segmental orientation (see supplemental Film S1 File for complete staining pattern). The combination of Gsb and Lbe patterns allows the identification of NB5-3 (yellow dotted lines) and NB5-6 (red dotted lines) cell clusters and shows NB5-3 cells in MN to lie very anterior.

The pattern of *gooseberry* expression (gsb-Gal4>UAS-CD4::tdGFP) helped with the identification of two further clone types in MN: Co-labelling with Ladybird early [41] unambiguously marks the NB5-3 lineage (Fig 9E) and reveals the position of these cells in the MN cortex (next to the NB5-2 cells). This enabled us to map a corresponding Flybow clone (Fig 9A and 9A´´). In MX and LB NB5-3 clones (Fig 9B and 9C´´) show an increasing degree of morphological conservation compared to the thoracic/abdominal homologs (Fig 9D).

**Fig 10:**
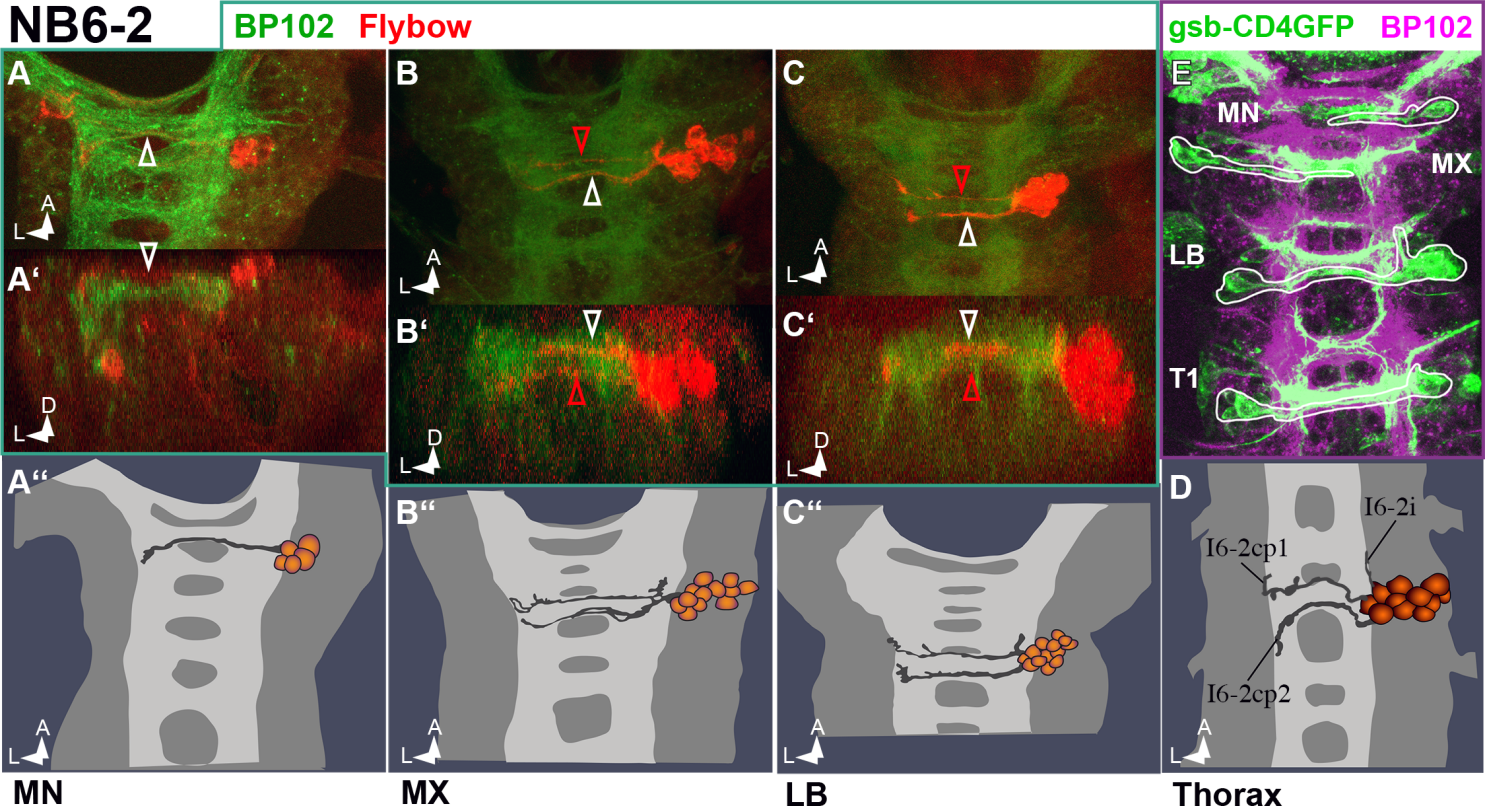
NB 6–2 lineage in gnathal segments. (**A–D)** Frontal (**A´, B´, C´**) and horizontal (all other) views of NB6-2 clones (semischematic drawings in **A´´, B´´, C´´** and **D** and composite confocal images in all other). In abdominal and thoracic segments (**D**) the NB6-2 lineage consists of dorsal interneurons sending two distinct fibre bundles through the posterior commissure and carrying a short ipsilateral ascending fascicle. This pattern is preserved in MX (**B–B´´**) and LB (**C–C´´**). Also the axonal positions within the commissure are similar, with the posterior bundle (white arrowhead) crossing dorsally and the anterior bundle (red arrowhead) ventrally. The smaller lineage in MN only generates the posterior bundle crossing dorsally (**A–A´´**). (**E**) Double-staining against BP102 (magenta) and gooseberry-GAL4>UAS-CD4::tdGFP (gsb-CD4GFP, green). Only dorsal layers are shown. The NB6-2 lineage (white lines) is part of the GFP pattern.

Because of its typical dorsal cell body positions the NB6-2 lineage can be identified within the gsb-Gal4>UAS-CD4::tdGFP pattern (Fig 10E) and appears present also in MN. Again both, the MN lineage size and projection pattern are reduced (Fig 10A and 10A´´), while the homologous clones in MX and LB (Fig 10B and 10C´´) match the thoracic/abdominal ones (Fig 10D).

**Fig 11:**
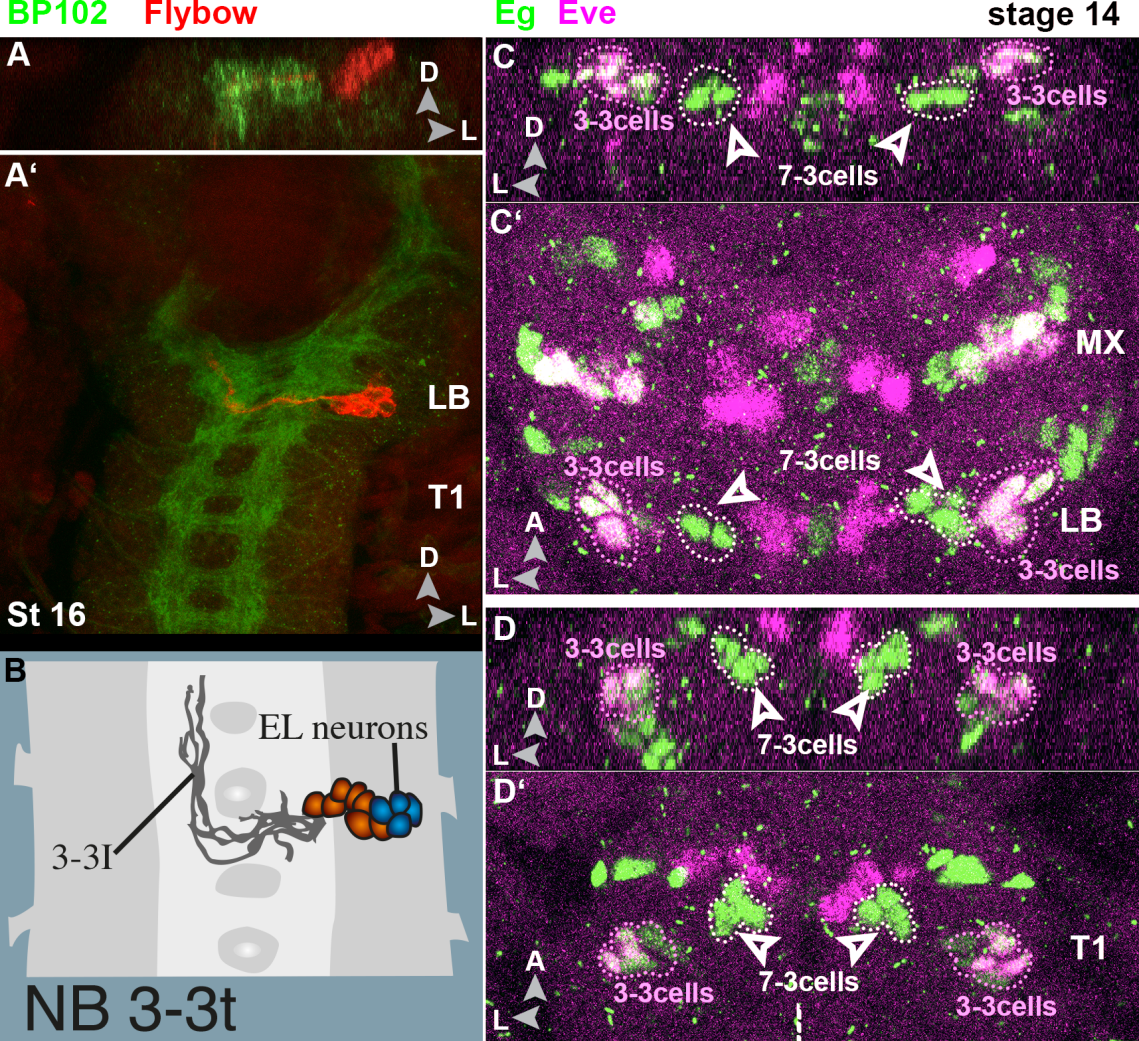
NB3-3 lineage in labial and maxillary segment. **(A-B)** Frontal (**A**) and horizontal (**A´**) view on a stage 16 ventral nerve cord showing a (potential) NB3-3 Flybow clone in the labial segment. The axonal morphology (A´) is very similar to the thoracic one (semischematic drawing of a NB3-3 DiI clone in B), but the cell bodies lie in a position dorsal to the neuropil (A). (**C-D**) Frontal **(C** and **D)** and horizontal **(C´** and **D´)** views on maximum projections of selected layers of stage 14 ventral nerve cords. NB3-3 progeny (encircled by pink dots) can be identified by co-expression of Even skipped (Eve, in magenta) and Eagle (Eg, in green). They lie lateral to the only Eg positive NB7-3 cells (white arrowheads) in all segments, but while they are more ventral than NB7-3 cells in T1 (**D**) they are more dorsal than NB7-3 cells in gnathal segments (**C**).

NB3-3 is formed in LB and MX, but lacking in MN [10]. Like in thoracic/abdominal segments NB3-3 progeny cells can be identified in LB and MX by the expression of Eagle (Eg, all cells) and co-expression of Eve in a subpopulation of cells (Eve-lateral cells, EL; Fig 11B–11D´ [14]). Their fibre projections are also similar to the thoracic ones (Fig 11A and 11B), but their cell bodies are located in a more dorsal position (Fig 11C and 11D).

For the identification of the NB1-1 lineage we made use of the molecular markers Even-skipped (Eve) and eve^RRK^-Gal4 (labelling a subset of Eve-positive cells; [19]). These markers show a stereotypic pattern in thoracic and abdominal segments, where they are co-expressed in the aCC motoneuron and the pCC interneuron (first progeny of NB1-1), and in the RP2 motoneuron (first progeny of NB4-2) [7,13]. However, in LB and MX, RP2 undergoes late apoptosis (stage 14/15), and in MN it is not formed (white arrowheads in Fig 4A and 4D; see also [10]). Furthermore, NB1-1 is not formed in MN (Fig 1 [10]) and in LB its daughter cell aCC undergoes apoptosis (stage 13–15; white arrows in Fig 4D). In MX the more dorsal cell of the aCC/pCC pairs (representing aCC in all other segments) is RRK- but not Eve-positive (inserts in Fig 4B and 4D)–an astounding observation having in mind that RRK is an enhancer-construct of the Eve locus [19]. Flybow clones reveal that the second motoneuron (1-1Ms) produced by thoracic NB1-1 [13,42] is lacking in the maxillary and labial lineages (like in abdominal lineages;), and that in LB the cluster of NB1-1 interneurons projects anteriorly and posteriorly (thoracic ones only posteriorly). Furthermore, in the late embryo (from stage 13) a number of Eve-expressing cells is found in MN and MX, which have no counterparts in more posterior segments (red arrows in Fig 4C and 4D and green arrows in Fig 4E).

From several NBs of which serial homologs have been identified in all three gnathal segments [10] (e.g. NBs 2–1, 3–1, 3–5, 4–1, 7–1) we obtained lineages in MX and LB that we could identity based on morphological similarity to more posterior segments. However, we were not able to identify corresponding lineages in MN (see Table 1), presumably due to highly derived characters. Some of these clones were labelled multiple times, revealing their reproducible composition (see Table 1 and S1 Fig).

We also compared the overall projection-patterns of the clones in the different segments. The percentage of clones that contain contralateral projections (MN: 69.4%, MX: 83.3%, LB 73.7%) showed no significant difference between the three segments. However, MN clones showed a significantly higher percentage of descending ipsilateral projections and a significantly lower percentage of ascending ipsilateral or contralateral projections, compared to MX or LB clones (Table 2). It remains to be investigated if this holds true also for the larval lineage morphologies and potentially reflects functional aspects of neuromere organisation.

**Table 2:** Percentage of clones with descending and ascending projections.

	MN	MX	LB
**ipsi des**	52.8	31.1	34.2
**ipsi as**	8.3	26.7	64.9
**cola as**	19.4	56.7	45.6

Ipsi des: ipsilateral descending; ipsi as: ipsilateral ascending; cola as: contralateral ascending. Light grey: significant; darker grey: highly significant.

Taken together, among the gnathal NB-lineages those in the MN show the highest degree of derivation compared to the ground state with regard to clonal sizes, projection patterns and marker gene expression.

## Discussion

Compared to the thoracic ground state the structures of the various CNS neuromeres are derived to different degrees. The three gnathal neuromeres, located between thoracic ventral nerve cord and posteriormost site (tritocerebrum) of the brain, form a specialized region (subesophageal zone; [27,43]), which is implicated in feeding and taste response (e.g., [44–49]). Structural composition of this region needs to be adjusted to these functional requirements.

The sizes of the gnathal neuromeres decrease from LB to MN, mainly due to smaller sizes of their neuroectodermal anlage (for fate maps see [50,51]) and thus lower amount of NBs to be formed. Comprehensive analysis of the embryonic NB-patterns in the gnathal segments revealed progressive reduction in NB-numbers from LB to MN [10], compared to the thoracic ground state[8]. The same trend is observed for the populations of postembryonic NBs. These NBs are in fact subsets of embryonic NBs that have been re-activated after a period of mitotic quiescence to undergo a second wave of proliferation in the larva [52]. Non-reactivated NBs undergo programmed cell death (PCD) at the end of embryogenesis ([53–55]. While in the thoracic neuromeres approximately 75% of the NBs become reactivated in the larva [32,38,56] it is only approximately 19% in the gnathal region [10,55]. Of the 14 NBs, which have been detected in the gnathal CNS of the late larva, eight to nine develop in LB and only approximately 6 in MX/MN. All of these NBs reside in the posterior segmental compartments [10,32,55].

In addition to decreasing numbers of NBs we find that their embryonic lineages in MN (see Table 1) generally consist of smaller numbers of cells compared to more posterior segments. Lineage sizes depend on control of proliferation and/or PCD. Proliferation can be controlled via the frequency of NB divisions and the time point of their cell cycle exit. Furthermore, NBs initially give rise to daughter cells (ganglion mother cells, GMCs), which divide once (type I, [57]) to produce two postmitotic progeny and, subsequently, most NBs switch to generating daughters that differentiate directly (type 0; [58–60]. For the ventral nerve cord it has recently been shown that abdominal segments exhibit decreased NB proliferation and an earlier type I>0 daughter proliferation switch as compared to thoracic segments [61]. While this leads to an A-P gradient of average lineage sizes decreasing in posterior (abdominal) direction [32,61], our data reveal a gradient of lineage sizes which in the gnathal CNS decreases in anterior direction (compared to thoracic ground state). PCD has been shown to occur in nearly all thoracic and abdominal NB-lineages [36], thus playing a major role in shaping these neuromeres. As demonstrated for a couple of identified cells (aCC, RP2), segment-specific PCD also occurs in the gnathal CNS (this study;[10]). Although we did not record PCD systematically, we assume that, in addition to proliferation control, PCD is a substantial source for size reduction in gnathal lineages.

In conclusion, the reduction of neuromere sizes (compared to ground state) from LB to MN is a result of decreasing numbers of embryonic (and postembryonic) NBs and decreasing sizes of their lineages.

Derivation of gnathal lineages from the ground state is also reflected by the expression of molecular markers.

The expression of Eve shows a stereotypic pattern in all thoracic and anterior abdominal segments [7,19]. This pattern becomes modified in the gnathal neuromeres not only by the absence of particular NBs and segment-specific PCD of specific progeny cells (aCC,RP2), but also by some Eve-expressing cells in MN and MX, which have no counterparts in more posterior segments. Wreden et al [62] recently reported that in the abdomen the Eve positive EL-neurons can be separated into two cohorts with late-born ELs contributing to a proprioceptive body posture circuit and early-born ELs being part of a mechanosensitive escape circuit. Since ELs are present in MX and LB and show a quite similar projection pattern (Fig 11A´), it will be interesting to a) test the share of both cohorts and b) identify the circuits they are part of. Detailed descriptions of the gnathal neuropil organisation [27,63] lay a good basis to search for potential input and output partners.

The expression of Eagle labels the entire lineages of NBs 2–4, 3–3, 6–4, and 7–3 in thoracic and abdominal segments [33,35]. In the gnathal CNS NBs 2–4, 3–3 and 6–4 are not formed in MN, and NB2-4 is also lacking in MX and LB [10]. Instead (from stage 12 onwards) further Eg-positive cell clusters were found in MN and MX (late progeny of NBs 6–2, 4–3, 4–4, 5–3, 5–6, and MNB) and in LB (progeny of NB5-3) [34]. Furthermore, serially homologous NB6-4 lineages diverge with regard to numbers and types of progeny cells: while the labial NB6-4 generates a mixed neuronal/glial lineage corresponding to the thoracic homologs, the maxillary NB6-4 gives rise to glial cells only, corresponding to the abdominal homologs [34]. A similar segmental correspondence is found for the NB7-3 lineage: while this lineage consists of three interneurons and one efferent GW neuron in both labial and thoracic (T1,2) segments, it consists of only three interneurons in maxillary and abdominal segments, and of only two interneurons in MN ([34], this study). In abdominal segments (and T3) the GW has been shown to undergo programmed cell death in the late embryo [36]. This is likely the case also for the GW in MX and MN, and for one interneuron in MN.

In addition to segment-specific absence of particular cells, axonal projection patterns are often modified. This is most obvious in MN, where a) only one commissure is present (Fig 2) and significantly more ipsilateral projections turn posterior and less ipsi- and contralateral projections turn anterior (Table 2). Due to these modifications, several of the gnathal (especially mandibular) NB-lineages could not be linked by morphological means to specific serially homologous counterparts.

We conclude, that in analogy to the derived numbers and expression profiles of gnathal NBs [10] the composition of the lineages of serially homologous NBs progressively differs from LB to MN.

Segment-specific differences in the composition of serially homologous lineages are under the control of Hox-genes, which have been shown to act at different levels of lineage development, including cell fate specification, cell division and programmed cell death (reviewed in [64]). While most studies focused on the thoracic and abdominal nerve cord, only little is known so far on Hox gene function in the developing gnathal CNS (e.g. [34,49,55,65]). Strikingly, while Hox genes are generally assumed to act in a cell-autonomous manner, an additional non-cell-autonomous function has recently been uncovered in the gnathal CNS for Hox genes of the *Antennapedia-Complex* [10]. Further candidate factors, possibly involved in patterning the gnathal CNS, have been identified by intersegmental comparisons of the combinatorial codes of marker gene expression. These comparisons not only revealed serial homologies among NBs based on similarities, but also modifications in their gene expression profiles, which progressively differ from LB to MN [34]. Since most of the marker genes considered by Urbach et al. (2016; [10]) encode transcription factors, their modified expression profiles likely have an impact on the composition of gnathal NB-lineages. Our analysis is in agreement with this assumption and provides a basis for investigations into the function of these factors in controlling the morphological characteristics of individual gnathal lineages.

## Supporting information

S1 Fig **Unidentified clone types A, B and C**.
**(A, B, C)** Frontal (**A´, B´ and C´**) and horizontal (all other) views of Flybow clones (semischematic drawings in **A´´, B´´** and **C´´,** composite confocal images in all other) showing repeatedly labelled types of lineages, we were not able to identify.
(**A–A´´**) Clone Type A. We found this clone 5 times in MX. It contains 6 to 12 cells that lie at the level of the neuropil. A contralateral projection runs dorsal and very anterior in the anterior commissure. At the lateral border of the ipsilateral connective a short projection is sent posteriorly.
(**B–B´´**) Clone Type B. This clone type was labelled 3 times in MN. It contains 9 to 10 cells in the dorsolateral cortex that project into the ipsilateral connective and turn posteriorly in a medial position.
(**C–C´´**) Clone Type C. Found 3 times in MN this clone has 6 to 11 cells in a ventral and medial position sending one bundle into the ipsilateral neuropil that turns posteriorly in a medial position.
(TIF)

S1 File Image Stack (as a movie) used for Fig 9E. Dorsal view on a stage 16 gsb-CD4GFP embryo that is stained for GFP (green), BP102 (blue) and Lbe (magenta). Because BP102 and Lbe antibodies are both from mouse they were detected with the same chromophore. But since their patterns do not overlap–BP102 stains neuropile fibres that are mostly dorsal; Lbe stains only few neuclei that are all located ventral to the neuropile–we separated the two stainings for clarity by colouring layers 1 to 18 blue and layers 19 to 51 magenta. NB5-3 and NB5-6 clusters of the different segments are highlighted be dashed lines.
(MP4)

S1 Table Comparing cell numbers of individual clone types between MN and more posterior segments. This table summarizes Mann-Whitney tests for all clone types we labelled in all three gnathal segments. P values revealing significantly smaller clone sizes in MN compared to MX+LB are on green ground.
(DOCX)
