# Supplementary figures and images for: Progressive derivation of serially homologous neuroblast lineages in the gnathal CNS of Drosophila
Source: PLoS One. 2018 Feb 7;13(2):e0191453. doi: 10.1371/journal.pone.0191453 (PMC5802887; doi:10.1371/journal.pone.0191453)

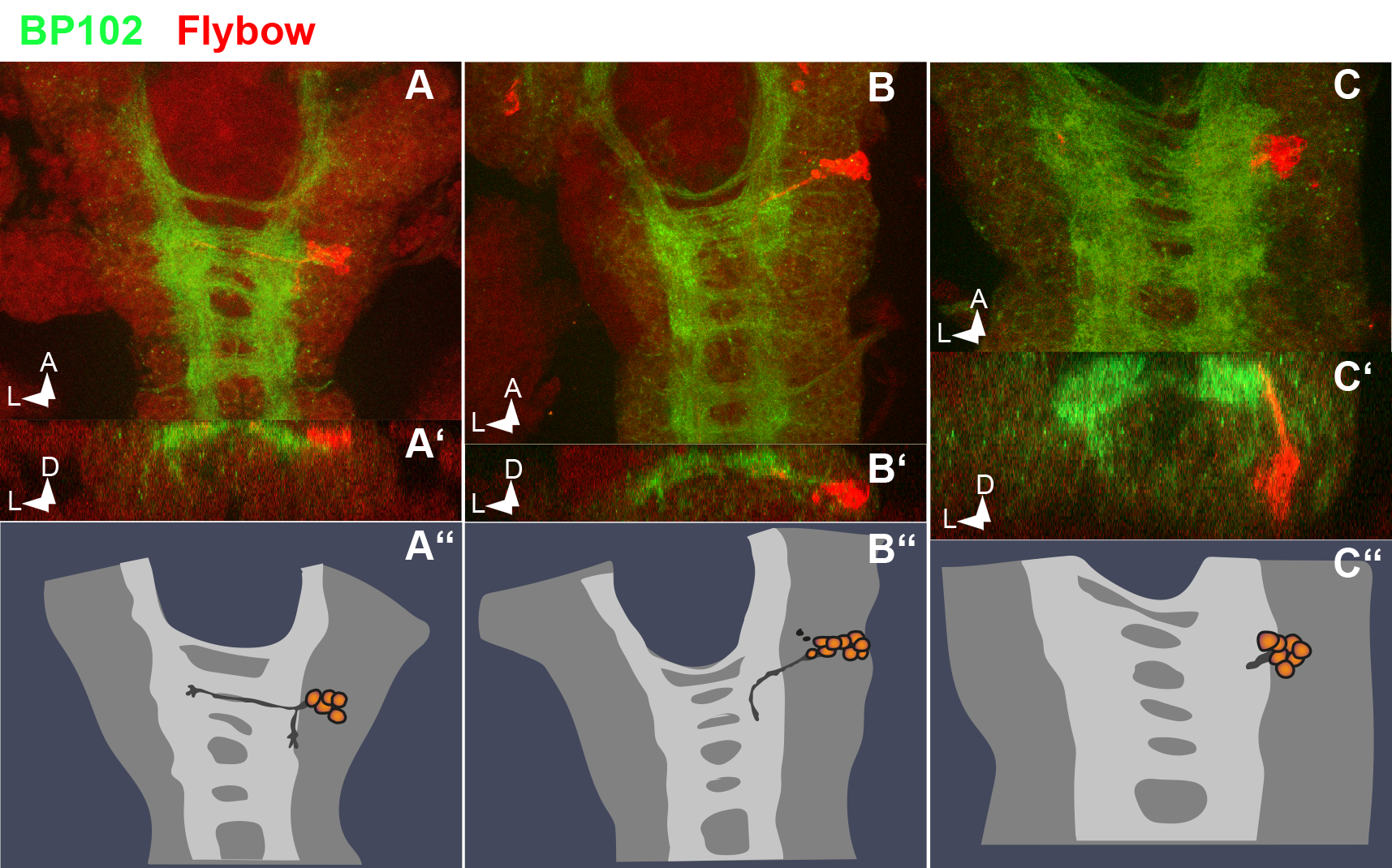

Supplement: S1 Fig — Unidentified clone types A, B and C. (A, B, C) Frontal (A´, B´ and C´) and horizontal (all other) views of Flybow clones (semischematic drawings in A´´, B´´ and C´´, composite confocal images in all other) showing repeatedly labelled types of lineages, we were not able to identify. (A–A´´) Clone Type A. We found this clone 5 times in MX. It contains 6 to 12 cells that lie at the level of the neuropil. A contralateral projection runs dorsal and very anterior in the anterior commissure. At the lateral border of the ipsilateral connective a short projection is sent posteriorly. (B–B´´) Clone Type B. This clone type was labelled 3 times in MN. It contains 9 to 10 cells in the dorsolateral cortex that project into the ipsilateral connective and turn posteriorly in a medial position. (C–C´´) Clone Type C. Found 3 times in MN this clone has 6 to 11 cells in a ventral and medial position sending one bundle into the ipsilateral neuropil that turns posteriorly in a medial position. (TIF) [file pone.0191453.s001.tif]
