# Supplementary material for: Progressive derivation of serially homologous neuroblast lineages in the gnathal CNS of Drosophila
Source: PLoS One. 2018 Feb 7;13(2):e0191453. doi: 10.1371/journal.pone.0191453 (PMC5802887; doi:10.1371/journal.pone.0191453)
Supplement: S1 Table — This table summarizes Mann-Whitney tests for all clone types we labelled in all three gnathal segments. P values revealing significantly smaller clone sizes in MN compared to MX+LB are on green ground. (DOCX) [file pone.0191453.s003.docx]

| **clone type** | **P value** | **number of clones in MN** | **number of cells per clone** | **number of clones in MX+LB** | **number of cells per clone** |
| --- | --- | --- | --- | --- | --- |
| **NB2-2** | 0.18 | 2 | 6, 7 | 6 | 11, 14, 4, 12, 9, 11 |
| **NB5-2** | 0.028 | 2 | 8, 9 | 11 | 20, 20, 20, 20, 14, 20, 20, 15, 9, 15, 16 |
| **NB5-3** | 0.373 | 1 | 8 | 5 | 12, 11, 10, 6, 12 |
| **NB6-1** | 0.035 | 2 | 3, 3 | 8 | 12, 19, 10, 14, 14, 16, 15, 10 |
| **NB6-2** | 0.115 | 1 | 5 | 7 | 19, 15, 16, 20, 19, 16, 19 |
| **NB7-2** | 0.013 | 6 | 5, 9, 8, 9, 8, 6 | 11 | 8, 14, 14, 9, 12, 17, 9, 8, 14, 14, 9 |
| **NB7-3** | 0.014 | 3 | 2, 2, 2 | 12 | 4, 4, 5, 5, 3, 4, 4, 4, 2, 3 |
